# Enteral Nutrition in Crohn’s Disease: A Comprehensive Review of Its Role in Induction and Maintenance of Remission and Perioperative Management in Adult Patients

**DOI:** 10.3390/nu17091481

**Published:** 2025-04-28

**Authors:** André Bargas, Carolina Palmela, Luisa Glória

**Affiliations:** Gastroenterology Service, Hospital Beatriz Ângelo, 2670-433 Loures, Portugal

**Keywords:** inflammatory bowel disease, Crohn’s disease, enteral nutrition, remission, maintenance

## Abstract

Crohn’s disease (CD) is a chronic inflammatory bowel disorder frequently associated with significant nutritional deficiencies. Enteral nutrition (EN), particularly exclusive enteral nutrition (EEN), has gained recognition not only for its nutritional support but also for its therapeutic potential in reducing intestinal inflammation. While EEN is well established in pediatric populations, its application in adults remains limited due to lower adherence and palatability challenges. Nonetheless, emerging evidence supports its efficacy in various clinical settings, including as an adjunct to pharmacologic therapies and in mitigating pre- and postoperative disease burden. The heterogeneity of study designs, formula compositions, and clinical protocols underscores the need for standardized guidelines and personalized approaches. This narrative review synthesizes the current evidence on the role of EN in adult patients with CD, with a focus on its use for induction and maintenance of remission, as well as perioperative optimization.

## 1. Introduction

Crohn’s disease is a chronic inflammatory bowel condition often complicated by malnutrition. The prevalence of malnutrition among individuals with inflammatory bowel disease (IBD) has been reported to range from 20% to 85% [1,2]. Malnutrition tends to become more prevalent as the disease progresses and is closely associated with its severity. Studies have shown that 39% of Crohn’s disease (CD) patients in remission exhibit signs of malnutrition, with this figure rising to 83% during active disease phases [3]. In a multicenter Spanish study, Casanova et al. [4] reported malnutrition in 16% of participants and the multivariate analysis revealed that previous abdominal surgery, active disease status, and food avoidance during disease flares were independently associated with an elevated risk of malnutrition [4]. This highlights the need for nutritional approaches in its management.

ESPEN defines enteral nutrition (EN) as all forms of nutritional support that involve the use of “dietary foods for special medical purposes”. This encompasses both oral nutritional supplements and tube feeding via nasogastric, nasoenteral, or percutaneous tubes [5]. In cases where it serves as the only form of nutritional support, it is called exclusive enteral nutrition (EEN). EN is classified into three main types based on the composition and degree of macronutrient hydrolysis. Polymeric formulas typically consist of 45% to 60% complex carbohydrates and polysaccharides, 15% to 20% intact protein, and 30% to 40% fat, primarily in the form of long-chain triglycerides. The main macronutrient sources in these formulations include corn syrup, soybean protein, and various plant-based oils such as canola and sunflower [6]. Due to the presence of intact macronutrients, polymeric formulas are generally more palatable and well-tolerated by individuals with intact gastrointestinal digestive and absorptive capacity. In contrast, semi-elemental formulas contain partially hydrolyzed proteins, typically in the form of oligopeptides, dipeptides, or tripeptides, along with medium-chain triglycerides, which facilitate digestion and absorption. Elemental formulas, on the other hand, are composed of fully hydrolyzed macronutrients, including free amino acids and simple sugars, and are characterized by a lower fat content, making them suitable for patients with compromised gastrointestinal function or severe malabsorption [6].

Diet has been implied in the pathophysiology of Crohn’s disease (CD), but the mechanisms by which EN exerts its therapeutic effects in Crohn’s disease are still being elucidated. One key mechanism involves modulation of the gut microbiota. EEN induces significant shifts in the intestinal microbiota, reducing the abundance of pro-inflammatory bacteria such as *Escherichia coli* while increasing beneficial species like *Ruminococcus gnavus*, *Ruminococcus torques*, and several Clostridium species [7,8]. Another study showed that responders to EEN exhibited a shift toward normalization of microbial and metabolic markers, including reduced levels of pro-inflammatory microbial metabolites such as trimethylamine and cadaverine, and increased abundance of beneficial butyrate-producing taxa like *Faecalibacterium prausnitzii* and *Roseburia* [9]. This microbial shift may contribute to its anti-inflammatory effects. These findings were also corroborated by recent studies, which demonstrated that the therapeutic effects of EEN in pediatric CD were closely linked to dynamic changes in the gut microbiota. EEN not only induced clinical remission but also drove individualized shifts in microbial composition and function. Notably, a reduction in microbial diversity during active disease, followed by enrichment of beneficial taxa such as *Lachnospiraceae*, was observed. In addition, medium-chain fatty acids (MCFAs) present in EEN formulas—such as lauric and decanoic acid—were shown to selectively activate protective bacterial species [10].

Furthermore, enteral nutrition (EN) has been shown to directly impact the immune response by downregulating pro-inflammatory cytokines and enhancing intestinal barrier integrity [11]. In vitro studies suggest that EN downregulates IL-6 and IL-8 production via the NF-κB and p38 MAPK pathways, mechanisms that are implicated in gut inflammation [12]. Additionally, EN has been associated with systemic changes that promote mucosal healing, including increased levels of insulin-like growth factor-1 (IGF-1) and transforming growth factor-beta (TGF-β1) [11].

EN, the provision of liquid nutrition formulas, has emerged as a significant therapeutic strategy, demonstrating both nutritional and anti-inflammatory benefits in Crohn’s disease management. EEN is particularly effective in inducing remission, especially in pediatric Crohn’s disease [6,13]. Clinical guidelines now recommend EEN as a first-line therapy for children with active Crohn’s disease due to its ability to achieve remission rates comparable to corticosteroids while avoiding their adverse effects [6,14].

In adult patients, the role of EEN is less well established, but it remains a viable option when corticosteroid avoidance is desired, according to the British Dietetic Association [15]. Beyond the induction of remission, there are several beneficial effects associated with EN. Partial EN has also been explored as a long-term strategy to maintain remission with studies suggesting that supplementing a normal diet with enteral formulas may reduce the risk of relapse in CD patients [16]. EN is also increasingly utilized in the perioperative setting for Crohn’s disease, as it has been shown to improve nutritional status and reduce postoperative complications [17]. Additionally, postoperative EN may help prevent disease recurrence. Despite its benefits, EN remains underutilized in clinical practice, particularly among adults.

The extensive variation in enteral formula composition and the uncertainty regarding its precise mechanisms of action contribute to the low use in clinical practice. While polymeric, semi-elemental, and elemental formulas have all demonstrated efficacy, no single formula has proved superiority in inducing remission in IBD [6,18]. Moreover, concerns regarding patient adherence and palatability limit the widespread adoption of EEN outside of the pediatric setting.

In summary, EN represents a valuable therapeutic strategy in CD. This review will examine the current evidence and explore the potential role of EN in CD management, including induction of remission, maintenance of remission, and in the perioperative setting.

## 2. Materials and Methods

An extensive literature search was conducted using the PubMed and Medline databases, from their inception to December 2024. The search strategy included Medical Subject Headings (MeSH) terms: (Crohn’s disease OR CD OR inflammatory bowel disease OR IBD) AND (enteral nutrition OR exclusive enteral nutrition OR EEN OR EN OR partial enteral nutrition OR PEN OR maintenance enteral nutrition OR MEN OR formula) AND (Adult). A supplementary manual search was also performed by reviewing the reference lists of the selected articles to identify additional relevant studies. Articles were screened for relevance to the scope of this narrative review. Studies of various designs were included, such as meta-analyses, systematic reviews, randomized controlled trials, observational studies, and retrospective studies.

## 3. Crohn’s Disease: Induction of Remission

### 3.1. Exclusive Enteral Nutrition

EEN refers to the administration of a nutritionally complete formula as the only source of nourishment. EEN is considered the primary therapeutic approach for achieving remission in children with mild to moderate Crohn’s disease. However, current clinical recommendations do not endorse its use as a first-line option for adults, as studies in adult CD populations have demonstrated comparatively lower effectiveness in inducing remission, ranging from as low as 20% to as high as 100% [19,20,21].

In pediatric CD, initial suggestions that newly diagnosed patients or those with ileal disease may respond more favorably to EEN were not corroborated by broader studies, which demonstrated efficacy across various disease phenotypes, including long-standing disease and those with ileocolonic or isolated colonic involvement [22].

A key challenge in implementing EEN in adult patients is the higher rate of non-adherence compared to pediatric cases, especially when the therapy is prolonged. Contributing factors include gastrointestinal side effects—such as bloating, flatulence, and diarrhea—as well as poor palatability, particularly due to the repetitive nature of the diet and the taste of the nutritional formulas. Due to these side effects, several studies report an intolerance rate needing diet discontinuation of around 20% [23].

In adults, early research indicated that EEN could be as effective as corticosteroids in achieving remission [24]. A controlled trial in 1984 involving 22 patients found that both steroid and elemental diet groups achieved an 80% remission rate at 4 weeks [24]. Notably, both groups experienced weight gain, both at the end of treatment and after three months, suggesting EEN’s additional benefit in active CD [24].

Similarly, Yamamoto et al. [25] showed that in 28 adult CD patients with active disease, undergoing EEN for 4 weeks, 71% achieved clinical remission. Endoscopic healing was reported in 44% of cases in the terminal ileum and 39% in the large bowel, while histologic healing was seen in 19% and 20%, respectively [25].

A broader review of adult CD patients, incorporating studies published between 1984 and 2002, found no significant difference between EEN and corticosteroids in achieving remission, though remission rates varied widely (20–100%) [21]. The primary challenge noted was adherence, with dropout rates reaching 41% for EEN, significantly higher than for corticosteroids (around 20%). No association was found between disease location and the likelihood of achieving remission with EEN or steroid therapy [21].

Recent prospective studies have further assessed EEN’s effectiveness. A prospective non-randomized pilot study in young adults with active CD [26] evaluated the efficacy of a two-week EEN regimen, followed by either 6 weeks of continued EEN or partial enteral nutrition (PEN). During the initial two-week EEN phase, the 32 participants experienced a significant reduction in Harvey-Bradshaw Index (HBI) scores (median decrease from five to three points, *p* = 0.003), serum CRP levels (median reduction from 10 to 5 mg/L, *p* = 0.005), and fecal calprotectin (FC) levels (median drop from 927 to 674 µg/g, *p* = 0.028). Additionally, median IGF-1 SDS levels showed improvement (from 0.0 to 0.05, *p* = 0.006). These improvements were sustained over the subsequent 6 weeks, supporting the role of EEN in inducing remission in adults [26]. Chen et al. [27] assessed the use of EEN combined with azathioprine in treating CD patients, including both adult and pediatric individuals with an average age of 28.9 years. Out of 29 participants, 23 (79%) experienced mucosal healing, with a mean duration of 123 days to reach this outcome, ranging between 50 and 212 days [27].

However, the results in the literature are contradictory. A 2018 systematic review reported a remission rate of 45% for EEN compared to 73% for corticosteroids, though the quality of evidence was low [28]. The study also found no significant differences in remission rates between elemental and non-elemental formulas. One of the conclusions of this study, as expected, was that patients on EEN were more likely to discontinue treatment due to adverse effects than those receiving corticosteroids.

Xu et al. [29] conducted a retrospective analysis aiming to explore factors that could potentially impact the effectiveness of EEN. Involving 91 patients, the study identified pancolitis as the most influential variable associated with treatment failure, followed by a low body mass index (BMI) [29].

EEN has also demonstrated efficacy in complicated CD. A study on 41 patients with fistulas, strictures, and abscesses found that 81% achieved clinical remission after 12 weeks of EEN (patients presenting with abscesses were additionally treated with antibiotics and drainage if needed), with 75% of those with enterocutaneous fistulas experiencing closure [30]. Another observational study in 48 adult CD patients with enterocutaneous fistulas reported a successful closure rate of 63% after three months [31], and in a study of 59 patients with inflammatory small bowel strictures, symptomatic remission was achieved in 81% also after three months, with radiologic and clinical remission rates of 54% and 65%, respectively [32]. Another retrospective study involving 31 adults [23] reported a notable improvement in both clinical manifestations and laboratorial markers following a median of 4 weeks of treatment in adults with complicated CD. This study reports that at the eight-week mark, clinical response rates varied across different CD phenotypes, with rates of 50% in B1, 79% in B2 and 100% in B3 [23].

A prospective, non-randomized study conducted in China investigated the impact of a 12-week EEN regimen in Crohn’s disease patients with active disease and complications [33]. Following the 12-week intervention, there was a significant reduction in the CDAI, with 81% of participants achieving full clinical remission. Among those with enterocutaneous fistulas, 75% experienced fistula closure and among those with stenotic disease, 20% of patients failed to respond to EEN and subsequently underwent surgery, while 20% achieved partial remission and 60% reached full remission. Additionally, resolution of intra-abdominal abscesses occurred in 76% of cases [33].

A systematic review assessing dietary interventions in fibrostenotic CD [34] found that approximately 60% of patients entered clinical remission following 12 weeks of EEN. Significant reductions in CRP (−63%) and bowel wall thickness (−59%) were reported. However, 15% of patients required surgery due to worsening obstructive symptoms, particularly those with fibrotic strictures. EEN appeared more effective for inflammatory strictures, whereas fibrotic strictures often necessitated surgical intervention despite dietary therapy [34].

EEN has also shown potential in patients with refractory CD. A retrospective study of six anti-TNF refractory patients found that EEN induced remission in 67% of cases after 12 weeks [35]. All patients showed improvement in disease activity scores (CDAI and PDAI) and inflammatory markers (ESR and CRP). Entero-enteric fistulas achieved complete radiologic remission, and perianal fistulas showed a significant reduction in drainage, though complete remission was not achieved. Clinical (BMI) and laboratorial nutritional parameters also improved. Notably, 75% of patients who achieved clinical remission were concurrently treated with anti-TNF agents, and all patients were under immunomodulators, reinforcing the potential benefit of combining EEN with biologic therapy [35].

Patients who achieve remission with EEN are typically continued on EEN for at least 6 to 8 weeks, followed by a structured reintroduction of whole foods. One suggested approach involves a gradual five-day transition, introducing one meal per day while tapering EEN intake [36]. Another strategy involves transitioning from EEN to maintenance PEN or other restrictive dietary regimens, such as the CDED, which is designed to mimic the effects of EEN by eliminating dietary triggers implicated in IBD onset and exacerbation.

In order to identify the most suitable candidates for EEN among adult Crohn’s disease patients, Wall et al. [37] conducted a study involving 38 adults between 16 and 40 years, either recently diagnosed or experiencing disease flares, who underwent either 8 weeks of EEN or a two-week course of EEN, succeeded by 6 weeks of PEN. The study assessed personality traits using the Big Five Inventory, revealing that adherence to EEN was significantly higher among individuals with greater conscientiousness scores [37]. These results indicate that patient personality traits may play a role in determining the success of dietary interventions, highlighting the importance of individualized treatment planning.

Despite challenges with adherence, particularly in adults, its benefits in clinical remission, mucosal healing, and nutritional improvement suggest that it remains a viable therapeutic option for managing CD, especially when corticotherapy or other treatments are unsuitable or poorly tolerated by patients. Table 1 summarizes the main studies on exclusive enteral nutrition for the induction of remission in Crohn’s disease.

### 3.2. Partial Enteral Nutrition

Partial enteral nutrition (PEN) is characterized by the consumption of 50% to 90% of caloric intake from formula, with the remaining calories derived from whole foods.

As previously discussed, a major limitation of enteral nutrition (EN) lies in the low adherence rates, largely attributable to the requirement of adhering to an exclusively liquid diet for a period of 6 to 8 weeks. Partial enteral nutrition (PEN) has been proposed as a strategy to mitigate this limitation by allowing the inclusion of a controlled proportion of specific solid foods, potentially enhancing patient compliance, treatment tolerability, and overall quality of life. There are some studies using PEN in the pediatric population, but few studies in adults. As an example, Levine et al. conducted a prospective clinical trial for 12 weeks, evaluating the effects of combining partial enteral nutrition (PEN) with the Crohn’s Disease Exclusion Diet (CDED) in pediatric patients with active Crohn’s disease (CD), in comparison to exclusive enteral nutrition (EEN) [38]. Both approaches led to high remission rates and reductions in inflammatory markers by week six, with no statistically significant differences between the groups. By week six, 75% (30/40) of children receiving CDED plus PEN achieved steroid-free remission, against 59% (20 out of 34) of those treated with EEN (*p* = 0.38). Notably, PEN combined with CDED demonstrated superior tolerance (98% vs. 74%, *p* = 0.002), as reflected by reduced withdrawal rates [38]. Johnson et al. [39] evaluated the use of PEN versus EEN in 50 children with active CD to investigate the role of a non-exclusive diet. The participants were randomly assigned to receive either 50% PEN or EEN. While both groups showed clinical and nutritional improvement, the PEN group exhibited a lower remission rate (15% compared to 42%) [39].

In a descriptive, uncontrolled study, Sigall-Boneh et al. [40] examined PEN combined with CDED in both pediatric and adult cohorts. The findings revealed that 70% of children and 69% of adults achieved clinical remission (Harvey-Bradshaw Index ≤ 3), with the normalization of elevated CRP levels in 70% of those in remission [40].

A 2018 prospective non-randomized clinical trial explored the effects of a two-week EEN regimen followed by either 6 weeks of continued EEN or a transition to PEN with the inclusion of one small meal of regular food. During the initial two-week EEN phase, the 32 participants experienced a significant reduction in HBI score, CPR, and FC levels. By week 8, outcomes between the EEN and PEN groups were comparable. The absence of significant differences between these two approaches was attributed to the initial two-week EEN phase before dietary reintroduction. Nonetheless, a key limitation of this study was the absence of a control group not receiving enteral nutrition [26].

A more recent study, conducted by Verburgt et al. [41] examined the impact of CDED with PEN or EEN-induced remission on gut microbiota composition over 12 weeks, compared with healthy controls. Their findings indicated a reduction in *Proteobacteria* abundance alongside an elevation in *Firmicutes*. Patients with CD exhibited two distinct metabotypes, M1 and M2, while all individuals in the healthy control group displayed the M1 metabotype. The M1 profile was characterized by elevated levels of Bacteroidetes and Firmicutes, reduced *Proteobacteria*, and higher small-chain fatty acid (SCFA) synthesis, while M2 was associated with increased Proteobacteria and SCFA degradation. Among patients who achieved remission through dietary intervention, the proportion classified as M1 increased progressively—from 48% at baseline to 63% by week 6, and reaching 74% by Week 12. These findings support the role of dietary strategies, such as PEN with CDED, in sustaining remission and highlight their potential as a more feasible long-term alternative to EEN in managing inflammatory bowel disease (IBD) [41].

In a systematic review conducted by González et al. [42], no differences were observed between EEN and PEN in terms of achieving high response rates of remission—clinical or analytical. Both PEN and EEN demonstrated similar outcomes, with remarkable success observed in patients following either regimen. This suggests that PEN demonstrates a comparable capacity to EEN in promoting remission among individuals with active Crohn’s disease. The authors further hypothesize that the predominant factor influencing the effectiveness of partial nutritional strategies is the nature of the accompanying dietary pattern. An important limitation in this paper is that the studies included had different diets combined with PEN, ranging from CDED to free diet [42].

Partial Enteral Nutrition emerges as a potentially effective alternative to EEN, offering better adherence and improved quality of life for patients, especially in the long term. While some studies show that PEN can achieve similar remission rates as EEN in Crohn’s disease, the effectiveness of this approach may largely depend on the diet that accompanies it. Therefore, rather than focusing solely on the method of enteral nutrition, the key factor in achieving optimal outcomes may lie in the specific dietary composition used alongside PEN.

## 4. Crohn’s Disease: Maintenance Remission

Studies refer to maintenance enteral nutrition (MEN) as the use of enteral nutrition formulas comprising at least a portion of the total caloric intake to maintain clinical remission in CD. Early studies in the 1990s demonstrated that MEN, either alone or combined with maintenance therapy, led to superior remission rates in comparison to maintenance therapy alone [43,44].

Between 2000 and 2010, six studies, including two randomized controlled trials (RCTs), evaluated MEN in conjunction with various treatments such as infliximab [45], steroids, azathioprine, and aminosalicylates. [46,47,48,49,50]. Five of these studies reported lower clinical relapse rates, often defined by a Crohn’s Disease Activity Index (CDAI) greater than 150, in patients receiving MEN. For example, Takagi et al. [49] conducted a comparative study assessing EN (n = 26) versus an unrestricted diet regimen (n = 25) as a maintenance approach alongside mesalamine or azathioprine in adult CD patients. Following a mean follow-up period of 11.9 months, clinical remission was maintained in 64% of patients receiving the supplemental elemental diet, in contrast to 35% among those adhering to an unrestricted diet (multivariate hazard ratio 0.4, 95% CI: 0.16–0.98) [49]. In contrast, one study found no statistically significant differences in clinical relapse rates between groups [45].

After 2010, four studies, including one RCT, investigated MEN in combination with treatments like aminosalicylates, sulfasalazine, 6-mercaptopurine [51], and anti-TNF agents [52,53,54]. In a study involving 74 consecutive CD patients, the concurrent administration of EN therapy—using either elemental or polymeric formulations—was recognized as an independent variable positively correlated with maintaining a prolonged therapeutic response to infliximab [52]. Similarly, a retrospective study with 102 adult CD patients found that the overall remission rate over time was markedly greater among patients receiving EN compared to those who did not receive such intervention (*p* = 0.009) [54]. Notably, Hanai et al. [51] found that MEN combined with these therapies resulted in improved clinical remission rates; however, Hirai et al. reported no significant differences in clinical remission when using MEN [53]. In most of these studies, the MEN dosage exceeded 35% of the caloric requirement.

A 2010 systematic review [55], using 10 studies mainly on Japanese patients (before the biologic era), concluded that although the overall quality of evidence was limited, EN appears to have potential utility in sustaining remission in patients with Crohn’s disease. Patients who received EN demonstrated a significantly greater rate of maintained remission compared to those who did not receive nutritional therapy. Moreover, the therapeutic effect of EN appeared to be dose-dependent, with higher volumes of enteral formula correlating with increased remission rates [55]. Another systematic review additionally showed that EN is superior to a regular diet and equally effective as select medications in sustaining remission in individuals with quiescent Crohn’s disease, concluding that MEN may be an attractive option for these patients [56].

The combination of EN with anti-TNF therapy has been explored to assess its impact on reducing the rate of development of secondary loss of response. Nguyen et al. [57] performed a meta-analysis and concluded that, in patients with moderate to severe Crohn’s disease receiving infliximab (IFX) therapy, the addition of enteral nutrition at a dosage of ≥600 kcal/day was associated with improved maintenance of clinical remission. Specifically, 69% of patients receiving specialized EN therapy with IFX achieved clinical remission, compared with 45% with IFX monotherapy [OR 2.73; 95% confidence interval (CI): 1.73–4.31, *p* < 0.01]. Similarly, 75% of patients on EN and IFX continued in clinical remission after 12 months, compared with 49% receiving IFX monotherapy [OR 2.93; 95% CI: 1.66–5.17, *p* < 0.01] [57]. Another meta-analysis in 2020 aimed to examine the therapeutic value of concurrent enteral nutrition and anti-TNF treatment. The maintenance of remission rate in the EN group was 71% (203/288), which was higher than 54% (306/569) in the non-EN group. The study concluded that the concomitant use of EN alongside anti-TNF therapy may contribute to the prevention of clinical relapse in patients with Crohn’s disease, including loss of response, during maintenance therapy. Although the optimal dosage of enteral nutrition remains undefined, several studies reporting clinical efficacy have cited intake levels of at least 600 to 900 kcal per day. It is noteworthy that most studies included in the review employed a retrospective cohort design, and high-quality prospective investigations remain limited. The optimal EN dosage, adherence levels, and timing for initiating EN to maximize combination efficacy remain unclear [58]. Table 2 summarizes the main studies on enteral nutrition for the maintenance of remission in Crohn’s disease.

## 5. Preoperative Setting: Enteral Nutrition

The role of EEN in the preoperative optimization of Crohn’s disease has gained increasing interest. While a more aggressive treat-to-target approach has reduced surgical rates, nearly half of all patients (47%) undergo surgery at some stage throughout the natural course of the disease [59,60]. The LIR!C trial demonstrated that, in patients with isolated ileocecal Crohn’s disease, primary surgical resection yielded quality of life outcomes at one year comparable to those achieved with medical therapy, supporting surgery as a viable first-line option in selected cases [61]. The European Crohn’s and Colitis Organization (ECCO) and the European Society of Coloproctology (ESCP) recommend considering early surgery for patients with penetrating or fistulizing disease, localized ileocecal disease, and obstructive symptoms without significant active inflammation [62]. Given this context, the need for strategies to optimize patients before surgery has become evident, and EEN has emerged as a potential intervention to improve outcomes by addressing both nutritional deficiencies and disease activity.

Nutritional deficiency has been strongly associated with an increased risk of adverse postoperative outcomes, as highlighted by the 2018 ECCO–ESCP Consensus on Surgery for Crohn’s Disease. [62]. This consensus recommends optimizing nutritional status before surgery through enteral or parenteral routes, as inadequate nutritional reserves can impair wound healing, increase infection risk, and prolong recovery time. The potential benefits of EEN in this setting are twofold: improving nutritional status, thereby reducing surgical complications and enhancing postoperative recovery, and reducing Crohn’s-related inflammation, which can lower the need for preoperative steroid use and optimize surgical outcomes.

Although the evidence for EEN as an inducer of remission is not strong, numerous studies and systematic reviews have provided supporting evidence for the advantages of preoperative nutritional optimization in the context of gastrointestinal surgical interventions, including CD [63,64,65]. A recent systematic review evaluated the evidence regarding the use of EEN for preoperative optimization in adult patients with Crohn’s disease subjected to intestinal resection. Seven retrospective studies were included—five cohort studies and two case-control designs. While EEN was associated with reductions in C-reactive protein and improvements in serum albumin, no consistent changes were observed in BMI or hemoglobin levels. A reduction in postoperative infectious complications was noted across studies, and there was a trend toward fewer stomas, although only one study demonstrated statistical significance. No differences in disease recurrence at 12 months were observed. Overall, one of the main limitations of this systematic review was the low to moderate quality and underpowered studies they used [66].

Several other studies have explored the benefits of EEN prior to surgery, providing further evidence of its role in preoperative optimization. A cohort study of 87 patients [67] found that in adult CD patients presenting with intra-abdominal abscesses, EEN was associated with a significantly lower rate of surgical intervention (26% vs. 56%, *p* = 0.01), and multivariate analysis confirmed EN as an independent predictor of reduced surgical risk [67]. Another retrospective study of 114 patients demonstrated reductions in CRP levels, fewer anastomotic leaks (2% vs. 18%; *p* = 0.023), and a lower need for temporary diverting stomas (23% vs. 41%; *p* = 0.036) with nutritional optimization [68]. Additionally, Heerasing et al. [69] followed 51 patients with complicated CD planned for surgery and found that, after preoperative EEN, surgical intervention was successfully avoided in 25% of patients [69]. The study also reported a ninefold decrease in postoperative abscesses and anastomotic leakage in the EEN group compared to controls, supporting its use as a preoperative intervention [69]. A large retrospective study by Li et al. [63] involving 497 patients divided into four groups—Group 1 (G1) consisted of patients who had not been exposed to immunosuppressive agents during the 8 weeks prior to surgery. Group 2 (G2) included patients who received immunosuppressive therapy up to the time of surgery, without a preoperative drug-free interval. Group 3 (G3) comprised patients who had a defined drug-free interval prior to surgery following immunosuppressant use. Finally, Group 4 (G4) consisted of patients who, in addition to a preoperative immunosuppressant-free interval, received EEN as part of their preoperative management. They found that those who received EEN preoperatively had better outcomes compared with the other groups, including lower rates of postoperative complications (G4 19% vs. G1 19%; G2 51.7%; G3 28.9%, *p* < 0.05; stoma formation (G4 18% vs. G1 22%; G2 66%; G3 34%, *p* < 0.05, as well as fewer urgent operations (G4 6% vs. G1 10%; G2 52%; G3 20%, *p* < 0.05) [63].

Costa-Santos et al. [70] conducted a prospective study to evaluate the effects of exclusive enteral nutrition (EEN) prior to intestinal resection in malnourished adults with complicated Crohn’s disease. They included patients over 18 years with evidence of active Crohn’s disease that was refractory to medical treatment and had a surgical indication due to the presence of a complicated disease phenotype, characterized by either stricturing or perforating manifestations. Ten patients received EEN for a median duration of 41.5 days. EEN was well tolerated in 83% of cases, with only two patients (17%) discontinuing. Significant improvements were observed in clinical and laboratory parameters: the Harvey-Bradshaw Index (HBI) decreased from 8.7 ± 1.9 to 4.1 ± 2.4 (*p* = 0.001), C-reactive protein (CRP) from 11.7 ± 10.3 mg/dL to 0.8 ± 0.8 mg/dL (*p* = 0.008), and serum albumin increased from 3.1 ± 0.6 g/dL to 4.0 ± 0.6 g/dL (*p* = 0.022). While BMI improved slightly, these changes were not statistically significant. Importantly, 20% of patients who received EEN no longer required surgery. When compared to the immediate surgery group (n = 5), there were no significant differences in postoperative outcomes, including complication rates (14% vs. 60%, *p* = 0.222), and hospital length of stay (8.4 ± 5.7 vs. 10.2 ± 4.9 days, *p* = 0.289). This group also analyzed microbiota, revealing that EEN induced significant changes in fecal microbial composition, particularly a reduction in alpha diversity and in Enterobacteriaceae abundance. However, these microbial shifts were not sustained long term, as diversity returned to near-baseline levels six months after surgery [70].

Steroid use is a risk factor for intra-abdominal septic complications, along with low albumin [71]. A prospective non-randomized study assessed 35 high-risk patients (defined as having abscesses or fistulas, stricturing disease associated with partial small bowel obstruction, or poor nutritional status—characterized by preoperative weight loss exceeding 10% of total body weight or the use of ongoing corticosteroid therapy) that received preoperative EEN. Discontinuation of steroids was achieved in 63% of patients (10/16), and postoperative morbidity rates did not differ significantly between high-risk and the controls, who were low-risk patients [72], suggesting that EEN can help bring high-risk patients to a more favorable baseline before surgery, potentially mitigating the impact of adverse prognostic factors.

A systematic review further reinforced that preoperative EEN significantly reduced inflammatory markers and was associated with lower rates of infectious and non-infectious complications [73]. Two studies even proved EEN to be an independent factor associated with a reduced risk of both infectious and non-infectious postoperative complications. Despite the results, three studies were considered “poor quality”, and only 1 fair quality [73]. Additionally, a retrospective study showed that EEN for penetrating CD allowed ileocolic resections with reduced incidence of postoperative complications [74]. These findings are further supported by a large Chinese review analyzing 708 surgeries, which examined four patient groups based on immunosuppressant exposure and EEN use (patients who had no prior exposure to immunosuppressive agents, patients who continued immunosuppressive therapy up until surgery, without any discontinuation period, patients who had a planned cessation of immunosuppressive drugs prior to the surgical procedure and patients who received 4 weeks of EEN via NG to a preoperative immunosuppressant-free interval regimen). Patients who combined a preoperative immunosuppressant-free interval with EEN had superior outcomes, including lower stoma creation rates and reduced postoperative infectious complications compared to those who did not receive EEN [63].

Wang et al. [33] also investigated the effects of preoperative EN on postoperative complications in CD. The study ensured that patients in both the EN and non-EN groups were well-matched regarding demographics, BMI, albumin, pre-albumin, hemoglobin, CRP levels, CDAI scores, smoking history, disease duration, prior surgeries, disease location, indications for resection, and medication history. The findings revealed that patients who received EEN preoperative experienced a significantly lower incidence of both infectious and non-infectious complications [33]. Notably, those who underwent 4 weeks of EEN therapy before surgery showed a reduced endoscopic recurrence rate at six months post-resection. However, by 12-, 18-, and 24-months post-surgery, Rutgeerts scores were comparable between the two groups (*p* > 0.05) [33].

The 2020 ECCO review of peri-operative nutritional interventions in IBD [75] acknowledges that EEN holds potential as a preoperative optimization strategy, contributing to both a reduction in postoperative complications and enhancement of patients’ nutritional status, but emphasized that the optimal duration and route of administration should be defined by a multidisciplinary team [75].

Despite these promising findings, the feasibility and adherence to EEN remains a challenge in clinical practice. A very recent three-arm RCT comparing EEN, phase 1 of CDED (half of the energy intake from oral PEN and the other half from specific foods), and standard of care for 6 weeks, in patients with >18 years who required elective gastrointestinal surgery to manage Crohn’s disease found that EEN had the lowest adherence (33%) due to psychological distress and difficulty maintaining the diet. Interestingly, CDED was well tolerated, with 80% of participants reporting high adherence (>90% of prescribed intake), in the absence of worsening gastrointestinal symptoms or notable weight loss. At week six, patients in the CDED group had significantly higher intake of energy and protein compared to standard care (*p* < 0.05). Although not powered to detect surgical outcome differences, the study demonstrated that preoperative phase 1 of CDED could be feasible and safe in the preoperative setting [76].

In summary, EEN appears as a promising preoperative strategy to optimize high-risk CD patients, reducing inflammation, improving nutritional status and post-surgical outcomes. While its benefits have been well-documented in several studies, challenges remain in its implementation, particularly in terms of patient adherence and the optimal duration of therapy. Further large-scale prospective studies are needed to establish standardized guidelines for its use in clinical practice, ensuring that patients receive the maximum benefit from this nutritional intervention while minimizing potential barriers to adherence. Table 3 summarizes the main studies on enteral nutrition in the preoperative setting.

## 6. Postoperative Setting: Enteral Nutrition

The influence of EEN extends beyond immediate surgical outcomes, impacting postoperative recovery and recurrence rates in CD. Postoperative enteral nutrition has been associated with a reduction in both clinical and endoscopic recurrence among CD patients [77,78].

Yamamoto et al. [78] conducted a study to assess the effect of EN on postoperative recurrence in CD patients. This study included 40 consecutive individuals who underwent resection for ileal or ileocolonic CD and were randomly assigned to receive either partial EN or a regular diet after surgery. At six months following surgery, endoscopic recurrence was identified in 25% of individuals in the EN group and in 40% among those receiving a conventional diet (*p* < 0.05). By the 12-month follow-up, recurrence rates had risen to 30% in the EN group and 70% in the non-EN group, suggesting a potential protective benefit of EN in preventing postoperative disease relapse (*p* < 0.05) [78].

A more recent single-center, randomized controlled trial conducted in 2024 [79] investigated the impact of EEN in high-risk CD patients following intestinal resection. A total of 84 patients were included to receive either azathioprine (AZA) alone or AZA in combination with a three-month course of postoperative EEN (AZA + EEN). Findings revealed that patients in the AZA + EEN group exhibited a markedly reduced incidence of endoscopic recurrence (ER) compared to those in the AZA-only group at 3 months (9% vs. 28%, *p* = 0.037) and 12 months (33% vs. 63%, *p* = 0.009) post-surgery. However, clinical recurrence rates at both 3 and 12 months were similar across both groups. At 3 months, patients in the AZA group reported higher Short Form-36 (*p* = 0.0003) and Inflammatory Bowel Disease Questionnaire (*p* < 0.0001) scores, reflecting better quality of life. However, from months 5 to 12, quality of life between the two groups became comparable. In conclusion, for high-risk CD patients, the addition of a three-month postoperative EEN regimen to AZA therapy appears to effectively reduce the 1-year ER rate, though it may transiently impact quality of life [79].

## 7. Conclusions

Enteral nutrition (EN) has emerged as a key therapeutic strategy in Crohn’s disease, with roles extending beyond nutritional support to active modulation of inflammation, gut microbiota, and mucosal healing. Its use spans multiple phases of disease management, including induction of remission, maintenance therapy, and perioperative care, with or without other therapies.

Remission rates in adult studies vary widely, and while some evidence suggests comparable efficacy to pharmacotherapy, the inconsistency in outcomes and higher dropout rates have prevented its adoption as standard therapy in this population. Despite limitations in study quality and heterogeneity of protocols, the collective evidence supports a broader integration of nutritional therapy—whether exclusive or partial—as part of a personalized treatment plan in Crohn’s disease management.

In the perioperative setting, EN has gained attention for its ability to optimize surgical outcomes. Preoperative EEN has demonstrated efficacy in enhancing nutritional status, reducing inflammatory burden, and decreasing the need for corticosteroids, ultimately leading to better postoperative recovery [69]. Studies suggest that preoperative EEN can significantly lower surgical complications, including anastomotic leaks and infections [63]. Similarly, postoperative EN appears to reduce clinical and endoscopic recurrence, particularly when combined with immunosuppressive therapy.

Some guidelines, such as those from Australia, recommend EEN in specific clinical scenarios where it offers distinct advantages. These include cases where corticosteroids are contraindicated or ineffective, when nutritional support is a primary concern (e.g., before semi-urgent bowel surgery), as an adjunct to other induction therapies like steroids or biologics, or in patients who are self-motivated and wish to explore a dietary approach instead of conventional IBD pharmacotherapies [36].

Despite its clear benefits, several challenges remain regarding the implementation of EN in routine clinical practice. High non-compliance rates, particularly among adults, highlight the need for strategies to enhance adherence, such as improved formula palatability, flexible dietary approaches, and better patient education [37]. Additionally, the optimal composition, duration, and delivery method of EN remain areas of ongoing investigation. While polymeric, semi-elemental, and elemental formulas have all demonstrated efficacy, patient preference and tolerability often dictate choice [6].

The authors suggest that polymeric formulas should be preferred for enteral nutrition in Crohn’s disease, as they are the most widely used, more frequently included in clinical studies, and generally better tolerated—leading to improved therapeutic adherence. Such formulas, including those enriched with TGF-β (Modulen^®^ IBD), are commonly used in clinical practice. Although no specific formula has demonstrated clear superiority [18], polymeric options remain the standard choice. Regarding duration, EEN is generally used for 2 to 8 weeks, with the authors recommending a minimum of 4 weeks when used as monotherapy to ensure clinical effectiveness.

In conclusion, EN represents a valuable and underutilized therapeutic option in Crohn’s disease, with evidence supporting its efficacy across various disease phases. Future research should focus on refining EN protocols, improving adherence strategies, and identifying patient subgroups most likely to benefit. Multidisciplinary approaches involving dietitians, gastroenterologists, and psychologists will be essential to maximize the therapeutic potential of EN and integrate it more effectively into personalized treatment strategies for Crohn’s disease.

## Figures and Tables

**Table 1:** Main studies of exclusive enteral nutrition in adult inflammatory bowel disease patients for induction of remission; EN enteral nutrition, EEN exclusive enteral nutrition, CD Crohn disease, CRP C-reactive protein, ESR erythrocyte sedimentation rate, PEN partial enteral nutrition, Alb Albumin, BMI body mass index, NGT naso gastric tube, CDAI Clinical disease activity index.

Author	Year	Study Type	Objectives	Methods	Population	Type of CD	Type of Nutritional Intervention	Efficacy Endpoints	Tolerance	Endpoint Values
Sanchit Sharma et al. [23].	2021	Retrospective	To evaluate the efficacy and tolerance of EEN in adults with complicated CD	31 adult patients received EEN as sole or adjunct therapy, followed up at 4 and 8 weeks	Adults only	Complicated CD: B1 (12.9%), B2 (58.1%), B3 (29%)	Exclusive enteral nutrition (74.2% semi-elemental, 25.8% polymeric); oral or via naso-enteric tube	Clinical response (CDAI reduction > 70), clinical remission (CDAI < 150), ↑ Hb and albumin	7/35 (20%) discontinued due to GI intolerance	Clinical Response: 80.4%; Clinical Remission: 16.1%; CDAI ↓ 290→186 (*p* = 0.001); Hb ↑ 7.7→9.8 g/dL (*p* = 0.001); Alb ↑ 2.5→3.2 g/d (*p* = 0.001);
Takayuki Yamamoto et al. [25]	2005	Prospective	Impact of elemental diet on mucosal inflammation and cytokines	28 adults received 4 weeks of elemental diet;	Adults only	Active CD: ileal (39%), colon (7%), ileocolonic (54%)	Elemental diet (Elental)	Clinical remission: CDAI < 150; endoscopic/histologic healing; ↓ cytokines; ↑ IL-1ra/IL-1β	All completed;	Clinical Remission: 71%; ↓ IL-1β, IL-6, TNF-α; ↑ IL-1ra/IL-1β (*p* < 0.05)
Catherine L. Wall et al. [26].	2017	Prospective	Assess feasibility/effects of EEN and PEN	38 adults: 2 w EEN then 6 w EEN or PEN;	16–40 yrs	Active CD; ileal or ileocolonic;	EEN→EEN or PEN (1 meal/day)	HBI, CRP, IGF-1, FC, albumin, BMI	14% intolerance to EEN after 2 weeks	First 2 weeks: HBI ↓ 5→3 (*p* = 0.003); CRP ↓ 10→5 (*p* = 0.005); FC ↓ 927→674 (*p* = 0.028); After 8 weeks of EEN: There was a sustained improvement in inflammatory biomarkers, with further improvement in the median HBI (*p* = 0.031)
Jia-Min Chen et al. [27].	2019	Prospective	Assess mucosal/transmural healing with oral EEN	29 CD patients; oral EEN;	Adults and children	Active CD: Ileal (38%), colonic (3%), ileocolonic (59%)	Oral EEN (Ensure + Peptisorb)	Mucosal healing: SES-CD ≤ 1; Transmural healing: bowel wall thickness ≤ 3 mm; CDAI, CRP.	All completed	Mucosal healing: 79%; Transmural healing: 17%; BWT: (9.41 vs. 4.97 mm, *p* < 0.001); SES-CD ↓ 14.9→0.9 (*p* < 0.001); CRP ↓ 24→3.3 (*p* < 0.001);
Yihan Xu et al. [29].	2019	Retrospective	Identify predictors of EEN response in isolated colonic CD	91 adults; predictive model built/validated using response outcomes	Adults only	Isolated colonic CD	EEN (TPF/Peptisorb); oral or NGT	Clinical remission (CRP < 10 and HBI ≤ 4).	7% of intolerance;	Clinical remission at primary cohort: 52.9%; Clinical remission at validation cohort: 47.4%
Qingfan Yang et al. [30].	2017	Prospective	Assess EEN in inducing remission in complicated CD	41 adults received 12 w EEN;	Adults only	Complicated CD: B2 (19.5%), B3 (80.5%)	EEN via NGT;	Clinical remission: CDAI < 150; Fistula/abscess resolution	5% intolerance	Clinical Remission: 80.5%; CDAI ↓ 223.4→106.8 (*p* < 0.001); Alb ↑, BMI ↑, CRP ↓ (*p* < 0.05) ¾ patients with ECF closed.
D. Yan et al. [31]	2014	Prospective	Identify predictors of EN response in CD with enterocutaneous fystula	48 adults received semi-elemental EN for 3 months	Adults only	Fistulizing CD with enterocutaneous fystula	Semi-elemental EN; via NGT	Fistula closure; CRP, ESR, BMI, Alb, Hb	Well tolerated overall	Fistula closure: 62.5% in 32 days; CRP ↓ 66.8→12 (*p* = 0.0003); Alb ↑ 39→43.9 (*p* = 0.0002); BMI ↑ 19→19.5 (*p* = 0.0004)
Dong Hu et al. [32]	2014	Prospective	Examine EEN efficacy in relieving inflammatory stricture	59 CD patients; 12 w elemental EN;	Adults only	Inflammatory stricturing CD	EEN (Peptisorb),via NGT;	Clinical remission: ↓ CDAI > 70;	84.7% completed full EEN	Clinical remission: 64.6%; CDAI ↓ 188→93 (*p* < 0.01); Alb ↑; BMI ↑ (*p* < 0.05)
Honggang Wang et al. [33].	2016	Retrospective	Assess pre-op EEN effect on postop complications and recurrence	81 adults: 42 received 4 w EEN, 39 controls;	Adults only	Ileal/ileocolonic CD with strictures	EEN (Peptisor), via NGT;	Post-op complications, recurrence;	100% completed	CRP ↓ 23.5→4.6, (*p* < 0.01); Alb ↑ 33.5→35.7, (*p* < 0.01); CDAI ↓ 213.9→139.8, (*p* < 0.01); Rutgeerts score 6 months post-operation, EN vs. Non-EN (3 vs. 10, *p* = 0.03)
Ajit Sood et al. [35].	2020	Retrospective	EEN in anti-TNF refractory adult CD	12 w oral semi-elemental EEN	Adults only	Ileocolonic CD: B3 (67%), B2 (33%)	EEN (Ensure Plus Peptide);	Clinical remission: CDAI < 150; fistula remission (complete cessation of fistula drainage)/response (reduction in fistula drainage of 50% or more)	100% completed;	Clinical remission: 66.7%; Fistula remission: 50% Fistula response: 100% CDAI ↓ 388.8→160.0 (*p* < 0.001);

**Table 2:** Main studies of enteral nutrition in adult inflammatory bowel disease patients for maintenance of remission; EN enteral nutrition, PEN partial enteral nutrition, CD Crohn disease, CRP C-reactive protein, NGT nasogastric tube, CDAI Clinical disease activity index, 6-MP 6-Mercaptopurine.

Author	Year	Study Type	Objectives	Methods	Population	Type of CD	Type of Nutritional Intervention	Efficacy Endpoints	Tolerance	Endpoint Values
Yamamoto et al. [45].	2010	Prospective non-randomized clinical trial	Assess EN with IFX in maintenance remission	56 patients post-IFX induction; EN + IFX (n = 32) vs. IFX only (n = 24)	Adult	Mixed	PEN: 50% of the energy from the EN	Clinical remission (CDAI < 150) at 56 weeks	22% stopped EN; no serious AEs	Remission: EN 78% vs. non-EN 67% (*p* = 0.51); no significant subgroup difference
Verma et al. [46].	2000	Prospective cohort	Assess PEN in maintaining remission	39 patients in remission; ED (n = 21) vs. control (n = 18); 12–24 months follow-up	Adults	Mixed	PEN ~35–50% daily intake	Remission at 12 months (CDAI < 150)	81% tolerated	Remission: 60% vs. 22% (*p* < 0.00001); Relapse: 33% vs. 77% (*p* < 0.00001)
Verma et al. [47].	2001	Randomized controlled trial	Assess PEN (polymeric vs. elemental) in steroid-dependent patients	33 steroid-dependent CD patients; ED (n = 19), polymeric (n = 14); 12 months	Adult	Mixed	PEN: Elemental/polymeric, 35–50% intake	Steroid-free remission (CDAI < 150)	82% tolerated	Steroid-free Remission: 43% both groups;
Takagi et al. [49].	2006	Randomized controlled trial	Evaluate ‘half EN’ as maintenance	51 patients’ post-remission, on mesalazine: half EN (n = 26) vs. free diet (n = 25); 28 months	Post-remission adults	L3 predominant	PEN: elemental formula 900–1200 kcal/day orally or via NGT	Relapses over 2 yrs (CDAI > 200)	77% tolerated	Relapse: 34.6% vs. 64.0%; HR 0.40 (95% CI: 0.16–0.98) (*p* = 0.04)
Yamamoto et al. [50].	2007	Prospective cohort	Evaluate long-term EN on clinical and endoscopic disease activity	40 CD patients in remission; EN (n = 20) vs. no NE (n = 20); 12-month follow-up with biopsies	Adults	Mixed	PEN with elemental formula: 1200–1800 mL/night	Clinical Relapse (CDAI ≥ 150);	90% tolerated	Clinical Relapse: 25% vs. 65% (*p* = 0.03); The mean endoscopic inflammation scores at 12 months were significantly elevated in the non-EN group compared to the EN group (*p* = 0.04);
Hanai et al. [51].	2012	Randomized controlled trial	Compare EN vs. 6-MP vs. control as maintenance (All with 5-aminosalicylic acid, 2250–3000 mg/day).	95 in remission; EN ≥ 900 kcal (n = 32), 6-MP (n = 30), control (n = 33)	Adult	L3 predominantly	PEN: elemental formula oral or NG ≥ 900 kcal/day	Clinical Remission at 24 months (CDAI < 200)	84% tolerated	Clinical Remission at 24 months EN vs. control: 46.9% vs. 21.2% (*p* = 0.0348); ED vs. 6-MP: 46.9% vs. 56.7% (*p* = 0.2733);
Sazuka et al. [52].	2012	Retrospective cohort	Identify clinical factors or concomitant therapies associated with sustained response to IFX.	74 CD patients on IFX; EN ≥ 600 kcal/day vs. control group < 600 kcal/day	Adult	Mixed	PEN: Elemental formula oral or via NGT	Loss of response (CDAI ≥ 150 + positive CRP)	Not evaluated	Loss of response: 20.6% vs. 52.3% (*p* = 0.0043);
Hirai et al. [53].	2022	Multicenter prospective cohort	Evaluate the effect of PEN with anti-TNF therapy	72 responders to IFX/ADA; EN ≥ 900 kcal/day (n = 37) vs. non-ED (n = 35); 2 yrs	Adult	L3 predominantly	PEN: Elemental formula oral or via NGT ≥ 900 kcal/day	Clinical remission (CDAI < 200) at 2 years	30% tolerated	Clinical Remission: 60.9% vs. 56.7% (*p* = 0.98);
Hirai et al. [54].	2013	Multicenter retrospective cohort	Evaluate the effect of PEN with anti-TNF therapy	102 CD patients; EN ≥ 900 kcal/day (n = 45) vs. non-EN (n = 57)	Adult	Mixed	PEN ≥ 900 kcal/day oral or NG	Recurrence: increase in CRP to >1.5 mg/dL or shortening of the IFX interval.	Not evaluated	Recurrence: 31.1% vs. 57.8% (*p* = 0.009);

**Table 3:** Main studies of enteral nutrition in adult inflammatory bowel disease patients in the pre-operatory setting; EN enteral nutrition, CD Crohn disease, CRP C-reactive protein, NGT nasogastric tube, BMI body mass index, HBI Harvey-Bradshaw Index, PN parenteral nutrition, TPN Total Parenteral Nutrition.

Author	Year	Study Type	Objectives	Methods	Population	Type of CD	Type of Nutritional Intervention	Efficacy Endpoints	Tolerance	Endpoint Values
Zheng et al. [67]	2017	Retrospective cohort study	To assess the impact of EN on risk of surgery in CD with intra-abdominal abscess	87 patients with spontaneous IAA; EN group (n = 23) vs. non-EN (n = 64); 4 weeks, median follow-up 1.9 yrs;	Adult	Ileal, colonic, ileocolonic, with intra-abdominal abscess	Elemental or polymeric EN 30–40 kcal/kg/day via tube	Need for surgery; Secondary: CRP, albumin,	Not evaluated	Surgery: EN 26.1% vs. non-EN 56.3% (*p* = 0.01); Albumin ↑ (*p* = 0.016), CRP ↓ (*p* = 0.002)
Guo et al. [68].	2016	Retrospective cohort study	Evaluate if pre-op nutritional therapy reduces leakage and stoma in CD	123 patients; NT group (n = 57); majority received EEN ~22.7 days	Adults	Ileal, colonic, ileocolonic; penetrating/stricturing	EEN (84%), EN + PN (10.5%), TPN (5.3%)	Primary: Anastomotic leakage; Secondary: diverting stoma	Not evaluated	NT vs. NNT Leakage: 2.3% vs. 17.9% (*p* = 0.023); Stoma: 22.8% vs. 40.9% (*p* = 0.036)
Heerasing et al. [69]	2017	Retrospective matched case–control	Evaluate if EEN reduce post-op complications in CD surgery	51 patients received EEN (13 avoided surgery); 38 analyzed vs. 76 matched controls ≥4 weeks	Adults	Ileal, ileocolonic, colonic with Stricturing/penetrating CD;	Oral EEN	Major complications (abscess/leak);	94% completed ≥4 weeks	Complications: 3% vs. 20% (*p* = 0.019)
Li et al. [63].	2015	Retrospective cohort	Evaluate if EEN reduce post-op complications in CD surgery	498 patients; 4 groups: G1 (n = 332) not exposed to immunosuppressive agents in the previous 8 weeks before surgery; Group 2 (n = 29) received immunosuppressive medications without preoperative drug-free interval; Group 3 (n = 128): preoperative immunosuppressants-free interval; Group 4 (n = 219): adding EEN to preoperative immunosuppressants-free interval regimen. EEN ≥ 4 weeks	Adults	Ileal, colonic, ileocolonic; Stricturing/penetrating CD;	EEN, via NGT	Infectious complications; Secondary: stoma, reoperation	Not evaluated	lower rates of postoperative complications (G4 19% vs. G1 19%; G2 51.7%; G3 28.9%, *p* < 0.05; stoma formation (G4 18% vs. G1 22%; G2 66%; G3 34%, *p* < 0.05, as well as fewer urgent operations (G4 6% vs. G1 10%; G2 52%; G3 20%, *p* < 0.05)
Costa-Santos et al. [70]	2020	Prospective observational	Clinical, nutritional and microbiota impact of EEN in surgical CD	15 CD patients referred to surgery: 12 eligible for EEN (median 41.5 days)	Adults	Ileal and ileocolonic Stricturing/penetrating CD	EEN	Primary: HBI, CRP, albumin, BMI, calprotectin; Secondary: complications	83% tolerated	HBI 8.7→4.1 (*p* = 0.001), CRP 11.7→0.8 (*p* = 0.008), albumin 3.1 to 4.0 (*p* = 0.022). Short term reduction in alpha diversity and in Enterobacteriaceae abundance
Beaupel et al. [72]	2016	Retrospective cohort study	Effect of polymeric diet enriched with TGF B2 on complications in high-risk CD surgery	56 patients: high-risk (n = 35) received Modulen^®^ ≥ 2 weeks pre-op (median 21.6 days)	Adults	Ileal and ileocolonic Stricturing/penetrating CD	EEN	Primary: complications; Secondary: steroids, stoma, feasibility	97% completed; well tolerated	Complications: 22.9% vs. 23.8% (*p* = 1.00); Stoma: 11.4% vs. 0% (*p* = 0.286)

## Abbreviations

The following abbreviations are used in this manuscript:

AZA	Azathioprine
BMI	Body Mass Index
CDAI	Crohn’s Disease Activity Index
CD	Crohn’s Disease
CRP	C-Reactive Protein
ECCO	European Crohn’s and Colitis Organization
EEN	Exclusive Enteral Nutrition
EN	Enteral Nutrition
ESR	Erythrocyte Sedimentation Rate
FC	Fecal Calprotectin
IBD	Inflammatory Bowel Disease
IBDQ	Inflammatory Bowel Disease Questionnaire
IFX	Infliximab
IL	Interleukin
MEN	Maintenance Enteral Nutrition
NGT	Nasogastric Tube
PEN	Partial Enteral Nutrition
RCT	Randomized Controlled Trial
SF	Short Form
TGF	Transforming Growth Factor
TNF	Tumor Necrosis Factor
